# CTM2‐2023‐06‐1111: Targeting regulatory T‐cells in pancreas during acute pancreatitis: Programmed‐death 1 blockage as a potential therapeutic for infectious pancreatic necrosis

**DOI:** 10.1002/ctm2.1472

**Published:** 2023-11-21

**Authors:** Deyu Zhang, Hua Yin, Chang Wu, Shiyu Li, Ghulam Nabi, Lisi Peng, Xiaotong Mao, Zhendong Jin, Zhaoshen Li, Xiaoju Su, Haojie Huang

**Affiliations:** ^1^ Department of Gastroenterology Changhai Hospital, Shanghai, People's Republic of China Shanghai China; ^2^ Department of Gastroenterology General Hospital of Ningxia Medical University, Ningxia, People's Republic of China Shanghai China; ^3^ Institute of Nature Conservation Polish Academy of Sciences Krakow Poland

Dear Editor,

Tregs play a pivotal role in infectious pancreatic necrosis. Treg‐activation disturbs the duodenal barrier function during severe acute pancreatitis and permits the translocation of commensal bacteria into pancreatic necrosis. The depletion of Tregs could decrease the bacterial translocation into pancreatic necrosis and targeting Tregs in acute pancreatitis (AP) may help to ameliorate the disease course.^1^ Here, we would like to highlight the important role of Programmed‐death 1+ (PD‐1+) pancreatic Tregs in infectious pancreatic necrosis after acute pancreatitis and propose a new potential therapeutic method through PD‐1 blockage.

First, to identify the symbolic change of immune cells between mild acute pancreatitis (MAP) and severe acute pancreatitis (SAP), we collected continuous peripheral blood samples from two MAP and one SAP patient and performed Cytometry Time Of Flight (Cytof) (Scheme map: Figure 1A, panel: Table S5). The two MAP patients were discharged after 10 days from the hospital without infection. The SAP patients were diagnosed with necrotizing pancreatitis and infectious pancreatic necrosis with fever and high procalcitonin(1.29 ng/ml) after 5 days in the hospital. The representative computed tomography (CT) image is shown in Figure S1A. Furthermore, the detected cells were divided into 30 subgroups (Figure 1B,C), and the main immune cell type is annotated in Table S5, based on the expression heatmap (Figure S1B). Among all the 30 subgroup clusters, the c12 (Treg) cluster was identified with a significant difference among different hospital times (Figure S1C and Figure 1D). A decreased proportion of Treg was seen in all three patients 1 day after onset. Then, the proportion of Treg was increasing in all three patients. Interestingly, the level of Treg continues to rise after ten days (Figure 1E), indicating the level of Treg may positively consist of compensatory anti‐inflammatory response and infectious pancreatic necrosis. Previous studies reveal that the existence of co‐inhibitory molecules is important for maturing and functionating of Treg.^2^, ^3^, ^4^ We next focused on the potential role of co‐inhibitory molecules in Treg in SAP. The main co‐inhibitory molecules include PD‐1, Programmed‐death ligand 1 (PD‐L1), PD‐L2, TIGIT, LAG3, and TIM3. PD‐1 is the only molecule expressed on Treg (Figure S1D), and the expression of PD‐1 on Treg shows the same tendency as the level of Treg (Figure 1F). Additionally, we perform single‐cell sequencing on normal mouse pancreas, mouse pancreas harvested at 4 h after inducing SAP, and mouse pancreas harvested at 18 h after inducing SAP (At this time point, mice are susceptible to infectious pancreatic necrosis). The UMAP plot was shown in Figure 1G (by group), and Figure S1E (by subgroup). The subgroup was annotated, and T cell was extracted (Figure 1H,I and Figure S1F). Then, Group 0 was identified as Treg through high expression of CD4, CD25 and FOXP3 (Figure 1J). Interestingly, PD‐1 (PDCD‐1) expression was highest in T cell, especially in Treg in samples at 18 hours after inducing SAP (Figure 1J,L and Figure S1G). Then, the proportion of Treg (group 0) in the pancreas was identified with decreasing after SAP immediately and a large increase at 18 hours after inducing SAP.

**FIGURE 1:**
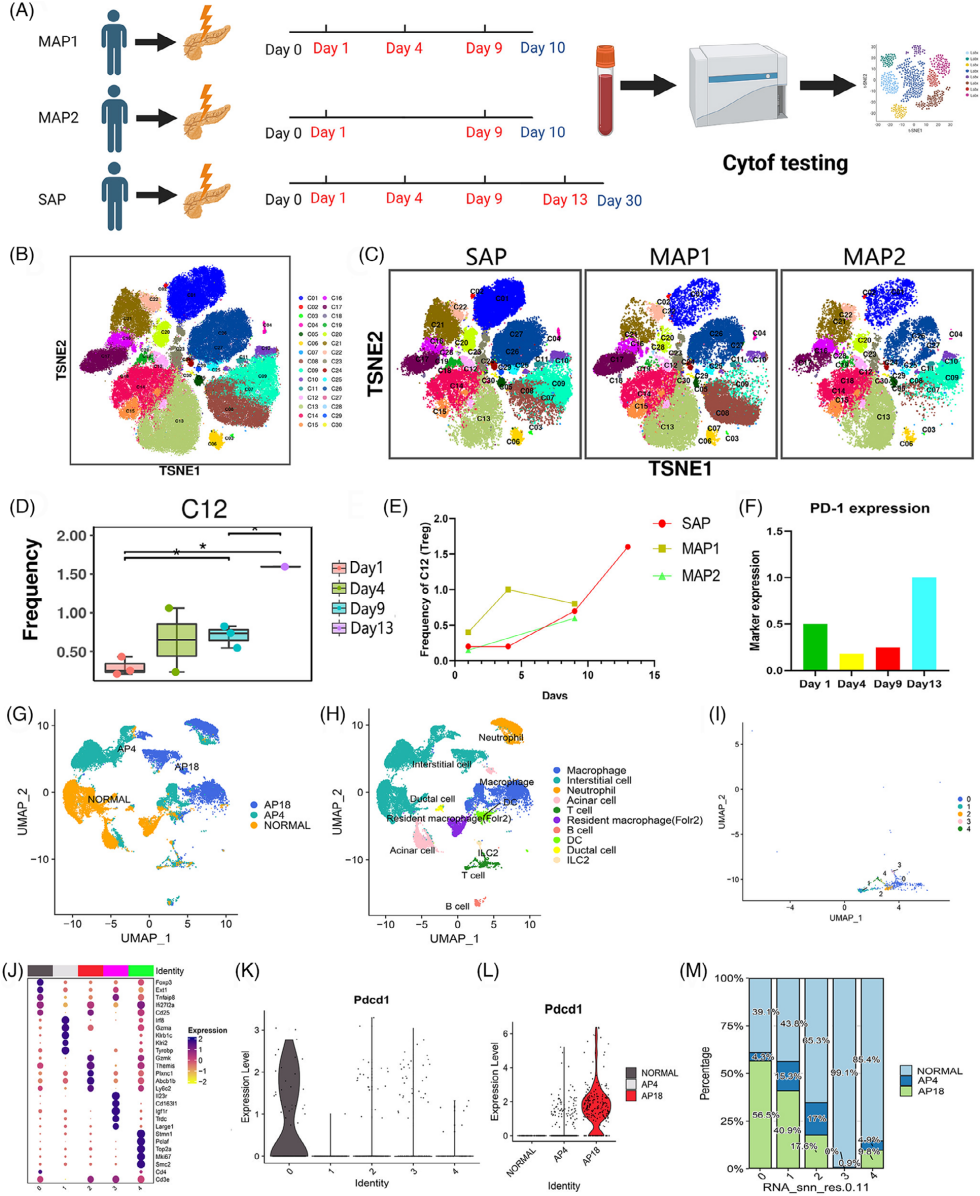
(A) Flow chart of our Cytometry Time of Flight (Cytof) testing. (B) TSNE map of all testing samples. (C) TSNE map of different patients. (D) The different frequencies of subgroup C12 (Treg) among different time points.* *p* < .05 (E) The different frequency of subgroup C12 (Treg) among different patients. (F) The expression of Programmed‐death 1 (PD‐1) on C12 (Treg) in severe acute pancreatitis (SAP) patients. (G) The UMAP plot shows the distribution of single‐cell sequencing data among the Normal group, AP4 group and AP18 group. (H) The UMAP plot shows the annotated cell group in the mouse pancreas. (I) UMAP plot shows the mouse pancreatic T cell can be divided into five cell subgroups. (J) The dot plot shows the expression of the top five genes, CD3 and CD4 in each cell subgroup in different pancreatic T cell subgroups. (K) The transcriptional expression of PD1 in different T cell subgroups. (L) The transcriptional expression of PD1 in group 0 among the normal group, AP4 group and AP18 group. (M) The proportion of different T cell subgroups among the normal group, AP4 group and AP18 group.

After identifying the high level of Treg and PD‐1 expression in the peripheral blood of infectious pancreatic necrosis patients and in the pancreas of a mouse model, we wondered whether these changes were sustained in the pancreas. Next, we designed a GFP‐marked bacteria infusion mouse model to stimulate infectious pancreatic necrosis after SAP and detected the expression of Treg (Foxp3) and PD‐1 (Figure 2A). According to the conclusion of a previous study, the proinflammatory cytokine reaches a peak after 12 h and the most severe necrosis is observed after 24 h,^5^ we set 9 and 21 h as the time point of systemic inflammatory response and compensatory ant‐inflammatory response (infectious pancreatic necrosis), respectively.

**FIGURE 2:**
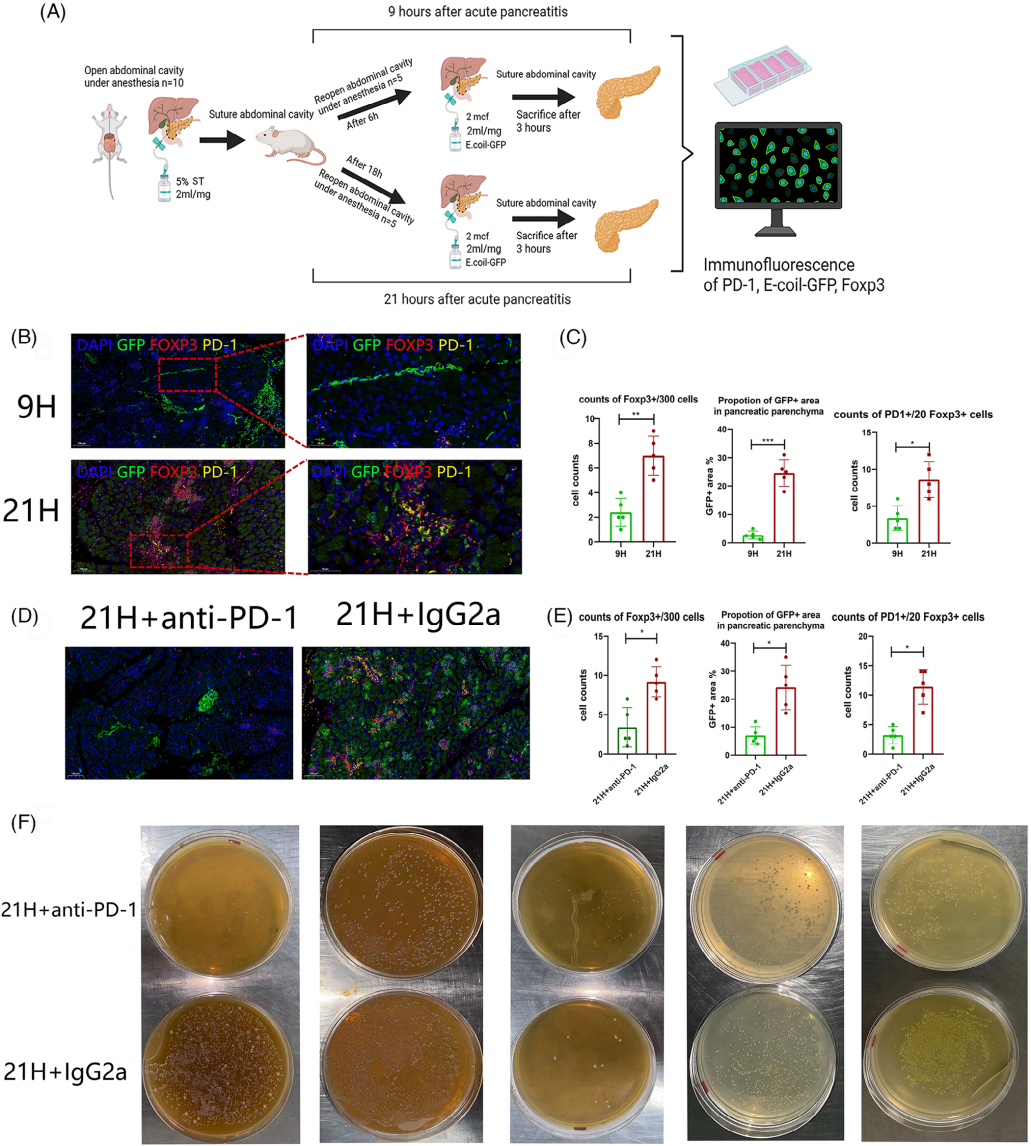
(A) Procedure of infectious pancreatic necrosis mouse model. (B) Immunofluorescence image of GFP (GFP marked E. coli bacteria), FOXP3 (Treg) and Programmed‐death 1 (PD‐1) on mouse pancreas between 9 h group (9H) and 21 h group (21H). (C) The different counts of Foxp3+ cells (Treg), PD1+ Foxp3+ cells and area of GFP+ area in pancreatic parenchyma between 9 h group (9H) and 21 h group (21H), * *p* < .05,** *p* < .01,*** *p* < .001. (D) Immunofluorescence image of GFP (GFP marked E. coli bacteria), FOXP3 (Treg) and PD‐1 on mouse pancreas between PD‐1 group (21H+PD‐1) and non PD‐1 group (21H+IgG2a). (E) The different counts of Foxp3+ cells (Treg), PD1+ Foxp3+ cells and area of GFP+ area in pancreatic parenchyma between PD‐1 group (21H+PD‐1) and non PD‐1 group (21H+IgG2a), * *p* < .05. (F) Result of spread plate using mixture of pancreas.

Intriguingly, most of the GFP‐marked bacteria were observed in the pancreatic duct or out of the pancreatic parenchyma in the 9 h group (9H) and they were dispersed in pancreatic parenchyma in 21 h group (21H), accompanied by the significant upregulated level of Treg and PD‐1 positive Treg (Figure 2B,C). Combined with the potential function of Treg reported by Glaubitz et al.,^6^ we hypothesized pancreatic PD‐1 expression and Treg facilitate the translocation of commensal bacteria into pancreatic necrosis. Then, we used PD‐1 antibody on a mouse model after 12 h to detect whether PD‐1 blockage therapy could reverse the bacteria translocation. Remarkably, the amount of GFP‐marked bacteria, PD‐1+ Treg and Treg significantly decreased (Figure 2D,E). The pancreas mixture's bacterial culture also supports this result. Specially, lower number of bacteria colony is identified in control group comparing to PD‐1 treatment group, suggection the improvement of infection in pancreas after PD‐1 therapy (Figure 2F).

Considering the reported rare complication about PD‐1 induced pancreatic injury,^7^ we detected the pathological score, lipase, and amylase levels between PD‐1 group(21H+PD‐1) and nonPD‐1 group(21H+IgG2a), and no significant changes were found between these two groups (Figure S2A,B).

Overall, inspired by the recent study related to Treg in acute pancreatitis and infectious pancreatic necrosis,^1^ we preliminarily further explored the expression level of Treg, put forward a novel mouse model of infectious pancreatic necrosis for the first time, and probe the potential therapeutic efficacy of PD‐1 blockage treatment in infectious pancreatic necrosis and the change in Treg. Our current data reveals the significant role of pancreatic Treg and PD‐1+ Treg in promoting translocation of commensal bacteria into pancreatic necrosis, PD‐1 blockage could be a novel therapeutic strategy for infectious pancreatic necrosis after acute pancreatitis. However, this is a preliminary study, and further study is needed to evaluate the impact of PD‐1 blockage in other immune cells in severe acute pancreatitis.

## AUTHOR CONTRIBUTIONS

Haojie Huang and Xiaoju Su contributed to the conception of the study. Hua Yin and Chang Wu collected the clinical samples. Deyu Zhang performed and visualized the experiment in silico. Deyu Zhang performed multiple immunofluorescence and wet experiments. Deyu Zhang and Ghulam Nabi performed the data analyses and wrote the manuscript. All authors contributed to the article and approved the submitted version.

## CONFLICT OF INTEREST STATEMENT

The authors declare no conflict of interest.

## FUNDING INFORMATION

This work was supported by the National Outstanding Youth Science Fund Project of the National Natural Science Foundation of China (No. 82022008), Major International Joint Research Programme (No. 82020108005), General program of the National Natural Science Foundation of China (No. 81770642) and General program of National Natural Science Foundation of China (No. 82170657).

## ETHICAL APPROVAL

The ethical approval was given by the Medical Ethics Committee of Changhai Hospital.

## Supporting information

Supporting Information

Supporting Information

Supporting Information

Supporting Information

## Data Availability

The data that support the findings of this study are available on request from the corresponding author, Haojie Huang, upon reasonable request.
