# Supplementary material for: CTM2‐2023‐06‐1111: Targeting regulatory T‐cells in pancreas during acute pancreatitis: Programmed‐death 1 blockage as a potential therapeutic for infectious pancreatic necrosis
Source: Clin Transl Med. 2023 Nov 21;13(11):e1472. doi: 10.1002/ctm2.1472 (PMC10660819; doi:10.1002/ctm2.1472)
Supplement: Supplementary file 1 — Supporting Information [file CTM2-13-e1472-s004.docx]

**Material and method**

**Patients**

This study included 2 MAP patients and 1 SAP patients who were hospitalized in the First Affiliated Hospital of Naval Medical University in 2022. Inclusion criteria: age ≥ 18 years, with a clear diagnosis of pancreatitis after blood biochemical and computed tomography (CT) examinations. All patients were classified according to the 2012 Atlanta Classification of AP^1^. The characteristic of patients is shown in Table S1. The study was approved by the Ethics Committee of the First Affiliated Hospital of Naval Medical University. All procedures performed in this study involving human participants were in accordance with the Declaration of Helsinki (as revised in 2013). For collection of serum samples, peripheral vein blood was collected from all recruited patients under a fasting state with vacuous tubes and then the red cells were cleared through bioGenous™ Red Blood Cell Lysis Solution (bioGenous, China), and the time point for blood collecting is shown in Figure 1A.

**Cytometry Time Of Flight (Cytof) and data acquisition**

The blood cells was centrifuged and the precipitated cell were washed once with 1xPBS and then stained with 100μL of 250nM cisplatin (Fluidigm) for 5min on ice to exclude dead cells, and then incubated in Fc receptor blocking solution before stained with surface antibodies cocktail for 30 min on ice. Cells were washed twice with FACS buffer (1xPBS+0.5%BSA) and fixed in 200μL of intercalation solution (Maxpar Fix and Perm Buffer containing 250nM 191/193Ir, Fluidigm) overnight. After fixation, cells were washed once with FACS buffer and then perm buffer (eBioscience), stained with intracellular antibodies cocktail for 30 min on ice. Cells were washed and resuspend with deionized water, adding into 20% EQ beads (Fluidigm), acquired on a mass cytometer (Helios, Fluidigm). The panel of the mass cytometry is shown as Table S2.

**Cytof data analysis**

Data of each sample were debarcoded from raw data using a doublet-filtering scheme^2^ with unique mass-tagged barcodes. Then, each .fcs file generated from different batches were normalized through bead normalization method^3^. FlowJo software was used to exclude to debris, dead cells and doublets, leaving live, single immune cells. Next, we apply the Phenograph clustering algorithm^4^ to all cells to partition the cells into distinct phenotypes based on marker expression levels. And then, cell type of each cluster was annotated according to its marker expression pattern on a heatmap of cluster vs marker, and the dimensionality reduction algorithm t-SNE^5^ was used to visualize the high-dimensional data in two dimensions and show distribution of each cluster and marker expression and difference among each group or different sample type. Finally, T-test statistical analysis was performed on the frequency of annotated cell population through R software.

**Preparation of the GFP marked bacteria**

GFP plasmid was bought from Addgene repository (Catalog: 48885, <https://www.addgene.org/48885/>). E. coli (DH5-alpha strain) was applied for cloning. For this aim, E. coli had been cultured overnight in LB broth. After achievement to 0.5 OD (in λmax = 600nm), it was centrifuged at 4°C for 15 minutes at 5000 RPM. Then, 1.5 ml of 100mM CaCl2 was added to the pellet for sensitizing. The suspension was placed on ice for 30 minutes. After the incubation period, it was centrifuged at 4°C for 15 minutes at 5000 RPM. Then, 400µl of 100mM CaCl2 was added to the pellet. A total amount of 2µl of plasmid was added on suspension and placed on ice for 30 minutes. The thermal shock was done for 42 seconds on 42°C and 2 minutes on ice, respectively. The transfected suspension was incubated on LB broth at 37°C. The transfected colonies, which grew on kanamycin contained LB agar was cultured on LB broth. Then, the concentration of GFP marked bacteria in LB was detected by Densimat densitometer (Cata log: WGZ-XT, Qiwei company, China), and the bacteria concentration was adjusted to 2 MCF using LB.

**Animal experiments**

8 weeks old male C57BL/6J mice (body weight 20 ± 1 g) were purchased from Beijing Vital River Laboratory Animal Technology Co., Ltd. and housed in individually ventilated cages (four animals per cage) at the SPF facility of Changhai hospital under controlled environmental conditions with free access to standard laboratory chow and tap water and were maintained on a regular 12/12 h light/dark cycle. All animal studies were approved by the Animal Experimentation Ethics Committee of Changhai hospital. Mice were acclimatized to their environment for 1 week before the experiments.

**Mouse model of severe pancreatitis (SAP)**

The procedure of severe acute pancreatitis (SAP) was used through sodium taurocholate-induced SAP, following the previous study^6^. Specifically, after the mice were anaesthetized with pentobarbital, laparotomy was performed and the duodenum was taken out gently from the depths of the wound in the middle of the abdomen. The biliopancreatic duct could be clearly observed by rotating the duodenum. The needle passed through the duodenal wall directly opposite to the papilla. Once the needle was placed within the duct, it was fixed in the desired position with a tied ligature. Retrograde infusion of 5% NaTc was performed at a flow rate of 40 µl/min, 2 µl/g according to mouse weight. After the retrograde infusion, the needle was removed and the opened duodenum was sutured. Mice were sacrificed at 4h or 18h post the initial induction of the sodium taurocholate-induced SAP (recognized as AP4 group and AP18 group, respectively), and the pancreas was collected for single-cell sequencing analysis.

**Tissue digestion**

Complete media was prepared with RPMI-1640 (Hyclone), 10% FBS (Gibco), and 1% penicillin-streptomycin (Hyclone). Mouse pancreas from mouse without SAP-inducing (Normal group, n=5), mouse pancreas from mouse at 4h post the initial induction of the sodium taurocholate-induced SAP (AP4, n=5), and mouse pancreas from mouse at 18h post the initial induction of the sodium taurocholate-induced SAP (AP18, n=5) were each minced with scissors and enzymatically digested in complete media supplemented with 1.0 mg/ml collagenase type IV (Sigma), 30 U/ml DNase type I (Sigma), and 0.5 mg/ml HAase type V (Sigma) for 50 min at 37 °C. Then the cells were filtered through the 70 μm cell strainers (Miltenyi Biotec), washed with phosphate-buffered saline (PBS), lysed in red blood cell buffer (BioTeke, China), and resuspended in PBS.

**Single-cell sequencing procedure**

According to the manufacture’s introduction, single-cell RNA-seq libraries were constructed using Single Cell 5′ Library and Gel Bead Kit. The libraries were sequenced using an Illumina Novaseq6000 sequencer with a sequencing depth of at least 77,618 reads per cell with pair-end 150 bp (PE150) reading strategy (performed by Oyi, Shanghai). Chromium Single Cell 3′ Reagent v3 kits were used to prepare libraries according to the manufacturer’s protocol. Single-cell suspensions were loaded onto the Chromium Single Cell Controller Instrument (10x Genomics, Pleasanton, CA, USA) to generate single-cell gel beads in emulsions (GEMs). After the generation of GEMs, reverse transcription reactions were performed. Then, cDNA was amplified, fragmented, end-repaired, A-tailed, index adapter ligated, and subjected to library amplification. Every library was sequenced on a NovaSeq 6000 platform (Illumina, San Diego, CA, USA), and 150-bp paired-end reads were generated. The Cell Ranger software pipeline (version 3.1.0) provided by 10x Genomics was used to demultiplex cellular barcodes, map reads to the genome and transcriptome using the STAR aligner, and downsample reads as required to generate normalized aggregate data across samples, producing a matrix of gene counts versus cells^7^.

**Data integration and annotation**

The matrix was input into Seurat pipline^8^. First, for each sample, gene expression matrices were normalized to the total cellular read count, and genes expressed in less than three cells and cell expressed less than 400 and more than 5000 genes were excluded. Then, the Seurat SCTransform function was applied to the normalized data to remove cell cycle effects and select 2500 highly variable genes (HVGs). Next, we checked HVG and removed mitochondrial genes from the HVG list. Next, we scaled and recalculated PCA for the cleaned HVG. The “RunUMAP” function was then applied to perform uniform manifold approximation and projection (UMAP) dimensional reduction. The “FindNeighbors” function was used to construct a shared nearest neighbor (SNN) graph, and the “FindClusters” function with the “resolution = 0.11” parameter was used to cluster major cells into different groups. The main cell types were identified on the basis of predicted and known marker genes acquired. Additionally, the “FindClusters” function with the “resolution = 0.21” parameter was used to cluster T cells into different group. In annotation of main celltypes, the total cell was divided into 18 subgroups (Figure S1E), and the expression of specific cell markers is shown in Figure S1F. The markers are chosen as previous study^9^, including Pdgfra and Col1a1 for interstitial cell, Ctrb1,Cpa1 and Try4 for acinar cell, Krt8 and Krt19 for duct cell, Pecam1 and Tek for endothelial cells, Mylk and Acta2 for pericyte, Ngp, S100a8/9 and Camp for neutrophil, Lyz2, Csf1r and Spp1 for macrophage, Cd3d and Cd3e for T cell, Cd79a and Igkc for B cell, Ncr1 and Nkg7 for NK cell, H2 Aa and Rel for dendritic cell, Il1rl1, Gata3 and Il7r for group 2 innate lymphoid cells (ILC2). The expression of there markers are shown in Figure S1F.

**Mouse model of infectious pancreatic necrosis**

The procedure of mouse model of infectious pancreatic necrosis is shown in Figure 2A. The key point of these procedure is based on retrograde injection of sodium taurocholate(ST) or the E.coil LB solution into the biliopancreatic duct. The procedure is based on the previous report^10^. Specifically, mice were randomly allocated into two groups (n = 5 for each group). All of the two group of mice endured retrograde injection of sodium taurocholate(2ml/mg) into the biliopancreatic duct to induce severe acute pancreatitis. Then, one group of mice endured retrograde injection of GFP-marked E.coli (2mcf, 2ml/mg) into the biliopancreatic duct after 6 hours to mimic the accumulation of bacteria in the pancreas. Another group of mice also endured retrograde injection of GFP-marked E.coli (2mcf, 2ml/mg) into the biliopancreatic duct after 18 hours. Finally, all the mice are sacrificed in 3 hours after the GFP marked E.coli injection, and then pancreas are collected in formalin solution at once.

**PD-1 antibody injection**

As for injection of PD-1 antibody, 10 mice were randomly allocated into two groups (n = 5 for each group): (1) anti-PD-1 antibody-injected group (21H+anti-PD-1, n=5) that received IP injections of invivomab anti-mouse PD-1 antibody (RMP1-14, Bio X Cell, West Lebanon, NH, USA) at 12 mg/kg in 6 hours after inducing acute pancreatitis; (2) Control mice group(21H+IgG2a, n=5) that received IP injections of the isotype control IgG2A antibody (2A3, Bio X Cell, West Lebanon, NH, USA) at 12 mg/kg in 12 hours after inducing acute pancreatitis. All of the two group of mice endured retrograde injection of sodium taurocholate (2ml/mg) into the biliopancreatic duct to induce severe acute pancreatitis. Finally, all groups of mice endured retrograde injection of GFP-marked E.coli (2mcf, 2ml/mg) into the biliopancreatic duct after 18 hours to mimic the accumulation of bacteria in the pancreas. Mice were sacrificed 3h after the last injection. Blood were collected for further measurements. Pancreatic tissue was isolated and divided into two parts. One parts are collected in formalin solution at once. Another parts of pancreas are grinded in sterile grinding bowl and resuspend in PBS solution (1mg/ml), respectively. And then, 10μL mixed solution are planted on kanamycin contained LB agar. The LB agar was photographed after 24 hours.

**Immunofluorescence staining**

For immunohistochemical staining, paraffin blocks of tissues originating from mouse pancreas were prepared. Paraffin-embedded pancreatic tissue sections were dewaxed and rehydrated twice in PBS for 15 min. The sections were incubated with 0.3% H2O2 for 15 min to block endogenous peroxidases, washed twice in PBS, incubated with 30 g/L BSA for 10 min to prevent nonspecific binding of antibodies, and then incubated with multiple antibodies (Table S3). Finally, diaminobenzidine (DAB) was added as a chromogen followed by hematoxylin. For immunofluorescence, the antibodies of cell markers involved in this procedure are listed in Table S2. Then, the slides of the paraffin block were incubated with multiple antibodies and counterstained with DAPI (catalog #ab228549, Abcam, USA). Finally, the slides were observed by a fluorescence microscope. T-test statistical analysis was performed on the counts of positive cells and markers between two groups.

**Serum amylase and lipase**

The blood was centrifuged (1500 rpm at room temperature) for 10 min. Serum was collected as supernatant. Then, amylase and lipase levels were immediately detected using the fully automatic biochemistry analyzer in the laboratory department of Changhai hospital in an hour. T-test statistical analysis was performed between two groups.

**Histopathology**

Pancreatic tissue was fixed in 10% neutral-buffered formalin, paraffin embedded and processed for histological analysis. Five-micron sections were stained with H&E and semiquantitatively scored using Schmidt’s criteria (Table S4) by two board-certified veterinary pathologists in a blinded manner. The final score is expressed as the average of these two values.

Supplemental Table 1. The characteristic of patients in this study

|  | Patients #1(MAP1) | Patients #2(MAP2) | Patients #1(SAP) |
| --- | --- | --- | --- |
| Severity | MAP | MAP | SAP |
| Age (years) | 35 | 43 | 37 |
| Etiology | Alcoholic | Alcoholic | Alcoholic |
| BMI (kg/m2) | 24.73 | 25.21 | 24.34 |
| Length of stay | 10 | 10 | 30 |
| Infectious pancreatic necrosis | None | None | Diagnosis at 5 day after onset. |

Supplemental Table 2. The marker panel of Cytof.

| Number | Marker | Number | Marker | Number | Marker | Number | Marker |
| --- | --- | --- | --- | --- | --- | --- | --- |
| 1 | CD45 | 12 | CD25 | 23 | CD33 | 34 | CD279_PD_1 |
| 2 | CD3 | 13 | CD274_PD_L1 | 24 | CD152_CTLA4 | 35 | CX3CR1 |
| 3 | CD56 | 14 | GranzymeB | 25 | CD278_ICOS | 36 | CD194_CCR4 |
| 4 | CD19 | 15 | CD39 | 26 | CD163 | 37 | CD223_LAG3 |
| 5 | TCRgd | 16 | CD366_TIM3 | 27 | CD185_CXCR5 | 38 | CD16 |
| 6 | CD196_CCR6 | 17 | Ki67 | 28 | T_bet | 39 | HLA_DR |
| 7 | CD14 | 18 | CD45RA | 29 | CD183_CXCR3 | 40 | CD4 |
| 8 | CD115 | 19 | CD86 | 30 | CD36 | 41 | CD8a |
| 9 | CD38 | 20 | CD27 | 31 | CD69 | 42 | CD11b |
| 10 | CD66b | 21 | CD197_CCR7 | 32 | CD273_PD_L2 | |  |
| 11 | TIGIT | 22 | CD11c | 33 | CD127 |  |  |

Supplemental Table 3. Antibodies involved in Immunofluorescence.

| Antibody Name | Company | Catalog No. | Region |
| --- | --- | --- | --- |
| GFP | Abconal | AE012 | China |
| FOXP3 | CST | D6O8R | USA |
| PD-1 | Abconal | A11973 | China |

Supplemental Table 4. Schmidt's score system of pancreatic histopathology.

| Pathologic changes | Scores | | | |
| --- | --- | --- | --- | --- |
|  | 0 | 1 | 2 | 3 |
| Inflammatory infiltrates | Absent | Mild | Moderate | Severe |
| Edema | Absent | Mild | Moderate | Severe |
| Parenchymal necrosis | Absent | Mild | Moderate | Severe |
| Haemorrhage | Absent | Mild | Moderate | Severe |

Supplemental Table 5. Annotated celltypes of each subgroup in Cytof.

| **Subgroup** | **Celltype** |
| --- | --- |
| C1 | NK cell |
| C2 | NK cell |
| C3 | Blood Platelet |
| C4 | Neutrophil |
| C5 | Basophilic Granulocyte |
| C6 | Neutrophil |
| C7 | Monocytes |
| C8 | Monocytes |
| C9 | Monocytes |
| C10 | Dendritic cell |
| C11 | Dendritic cell |
| C12 | Treg cell |
| C13 | central memory CD4 T cell |
| C14 | CD4+ T cell |
| C15 | Th1 cell |
| C16 | memory CD8+ T cell |
| C17 | central memory CD8+ T cell |
| C18 | CD4+ T cell |
| C19 | Tfh cell |
| C20 | activated CD8+ T cell |
| C21 | activated CD8+ T cell |
| C22 | CD8+T cell |
| C23 | Th2 cell |
| C24 | B cell |
| C25 | B cell |
| C26 | B cell |
| C27 | B cell |
| C28 | Tfh cell |
| C29 | B cell |
| C30 | Monocytes |


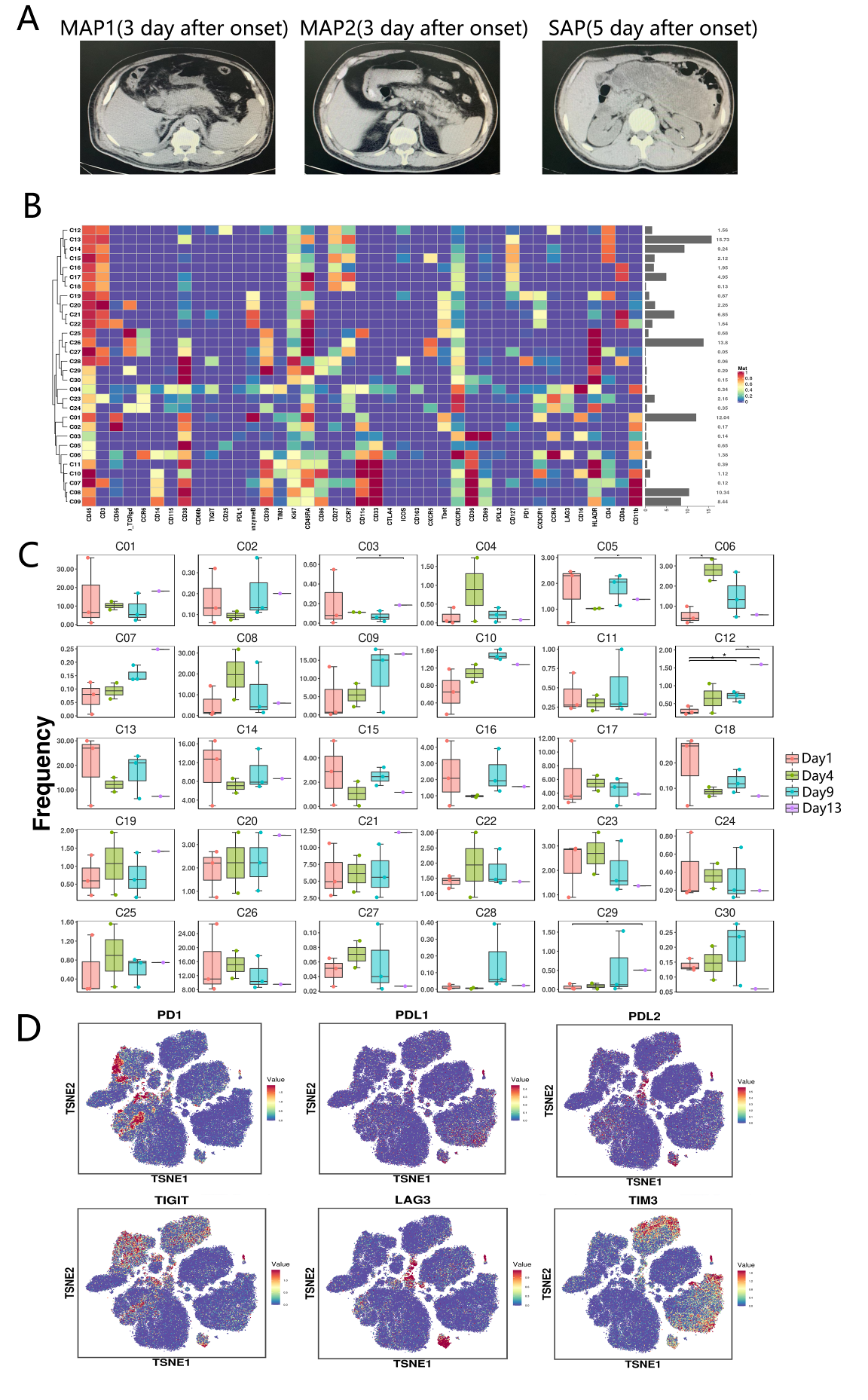


Figure S1. (A) The representative CT imagine of each enrolled patients.

(B) The scaled heatmap of each markers in each subgroup. (C) The proportion of each cluster among different time point, * P<0.05. (D) The TSNE distribution of six main co-inhibitory molecule.


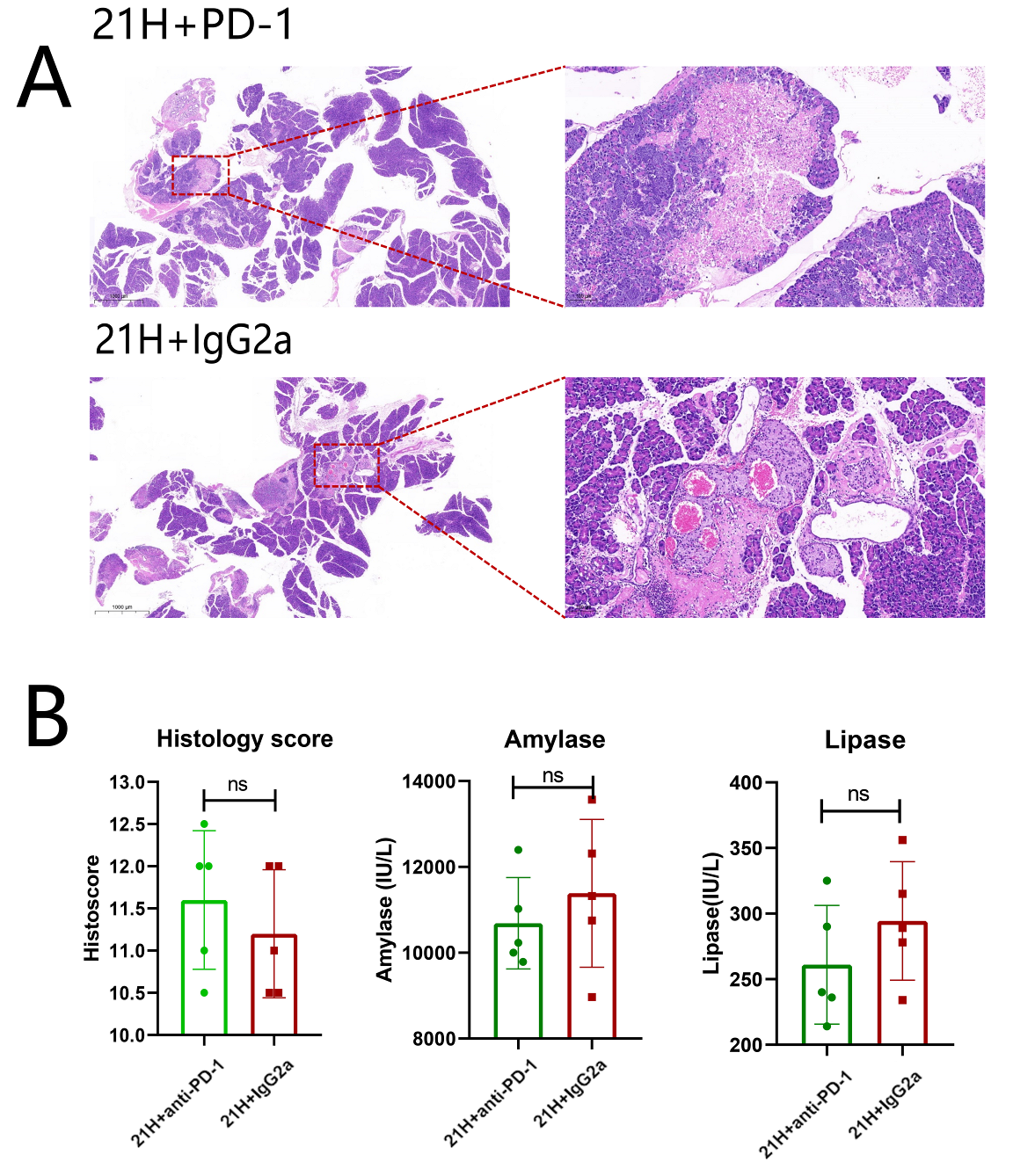


Figure S2 (A) The representative HE imagine of mouse pancreas between 21H+PD-1 group and 21H+IgG2a group. (B) The histology score, amylase and lipase level between 21H+PD-1 group and 21H+IgG2a group, ns P > 0.05 (no significant).

**Reference**

1. Banks P, Bollen T, Dervenis C, et al. Classification of acute pancreatitis--2012: revision of the Atlanta classification and definitions by international consensus. *Gut.* 2013;62(1):102-111.

2. Zunder ER, Finck R, Behbehani GK, et al. Palladium-based mass tag cell barcoding with a doublet-filtering scheme and single-cell deconvolution algorithm. *Nature protocols.* 2015;10(2):316-333.

3. Finck R, Simonds EF, Jager A, et al. Normalization of mass cytometry data with bead standards. *Cytometry Part A : the journal of the International Society for Analytical Cytology.* 2013;83(5):483-494.

4. Levine JH, Simonds EF, Bendall SC, et al. Data-Driven Phenotypic Dissection of AML Reveals Progenitor-like Cells that Correlate with Prognosis. *Cell.* 2015;162(1):184-197.

5. Zhou B, Jin W. Visualization of Single Cell RNA-Seq Data Using t-SNE in R. *Methods in molecular biology (Clifton, NJ).* 2020;2117:159-167.

6. Chen S, Zhu J, Sun LQ, et al. LincRNA-EPS alleviates severe acute pancreatitis by suppressing HMGB1-triggered inflammation in pancreatic macrophages. *Immunology.* 2021;163(2):201-219.

7. Zheng GXY, Terry JM, Belgrader P, et al. Massively parallel digital transcriptional profiling of single cells. *Nature Communications.* 2017;8(1):14049.

8. Butler A, Hoffman P, Smibert P, Papalexi E, Satija R. Integrating single-cell transcriptomic data across different conditions, technologies, and species. *Nature biotechnology.* 2018;36(5):411-420.

9. Yang X, Chen J, Wang J, et al. Very-low-density lipoprotein receptor-enhanced lipid metabolism in pancreatic stellate cells promotes pancreatic fibrosis. *Immunity.* 2022;55(7):1185-1199.e1188.

10. Zhou X, Chen H, Wei X, He Y, Xu C, Weng Z. Establishment of a Mouse Severe Acute Pancreatitis Model using Retrograde Injection of Sodium Taurocholate into the Biliopancreatic Duct. *Journal of visualized experiments : JoVE.* 2022(182).
