# Supplementary figures and images for: CTM2‐2023‐06‐1111: Targeting regulatory T‐cells in pancreas during acute pancreatitis: Programmed‐death 1 blockage as a potential therapeutic for infectious pancreatic necrosis
Source: Clin Transl Med. 2023 Nov 21;13(11):e1472. doi: 10.1002/ctm2.1472 (PMC10660819; doi:10.1002/ctm2.1472)

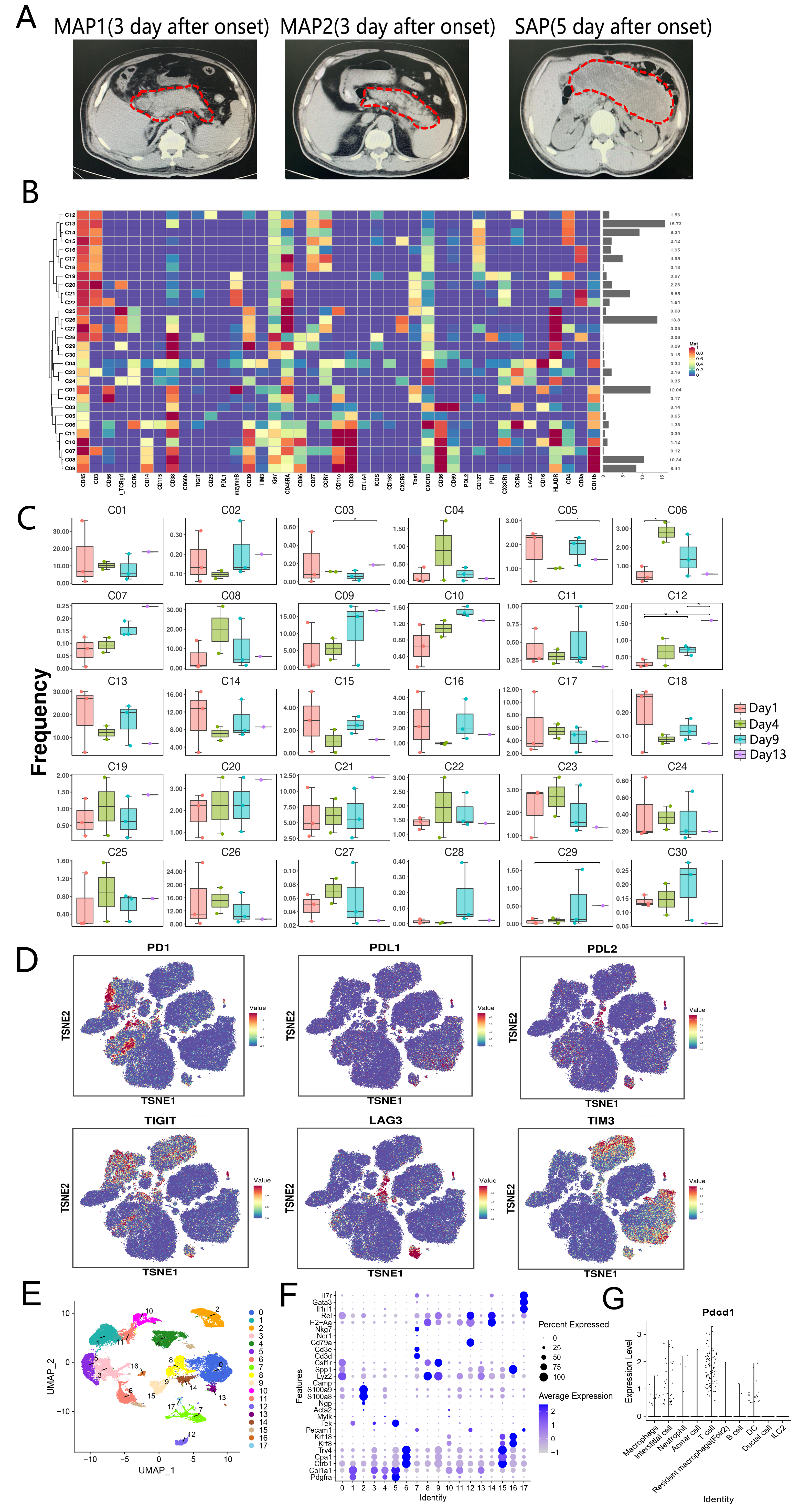

Supplement: Supplementary file 2 — Supporting Information [file CTM2-13-e1472-s001.tif]
