# Supplementary material for: CTM2‐2023‐06‐1111: Targeting regulatory T‐cells in pancreas during acute pancreatitis: Programmed‐death 1 blockage as a potential therapeutic for infectious pancreatic necrosis
Source: Clin Transl Med. 2023 Nov 21;13(11):e1472. doi: 10.1002/ctm2.1472 (PMC10660819; doi:10.1002/ctm2.1472)
Supplement: Supplementary file 4 — Supporting Information [file CTM2-13-e1472-s003.docx]

Supplemental Table 1. The characteristic of patients in this study

|  | Patients #1(MAP1) | Patients #2(MAP2) | Patients #1(SAP) |
| --- | --- | --- | --- |
| Severity | MAP | MAP | SAP |
| Age (years) | 35 | 43 | 37 |
| Etiology | Alcoholic | Alcoholic | Alcoholic |
| BMI (kg/m2) | 24.73 | 25.21 | 24.34 |
| Length of stay | 10 | 10 | 30 |
| Infectious pancreatic necrosis | None | None | Diagnosis at 5 day after onset. |

Supplemental Table 2. The marker panel of Cytof.

| Number | Marker | Number | Marker | Number | Marker | Number | Marker |
| --- | --- | --- | --- | --- | --- | --- | --- |
| 1 | CD45 | 12 | CD25 | 23 | CD33 | 34 | CD279_PD_1 |
| 2 | CD3 | 13 | CD274_PD_L1 | 24 | CD152_CTLA4 | 35 | CX3CR1 |
| 3 | CD56 | 14 | GranzymeB | 25 | CD278_ICOS | 36 | CD194_CCR4 |
| 4 | CD19 | 15 | CD39 | 26 | CD163 | 37 | CD223_LAG3 |
| 5 | TCRgd | 16 | CD366_TIM3 | 27 | CD185_CXCR5 | 38 | CD16 |
| 6 | CD196_CCR6 | 17 | Ki67 | 28 | T_bet | 39 | HLA_DR |
| 7 | CD14 | 18 | CD45RA | 29 | CD183_CXCR3 | 40 | CD4 |
| 8 | CD115 | 19 | CD86 | 30 | CD36 | 41 | CD8a |
| 9 | CD38 | 20 | CD27 | 31 | CD69 | 42 | CD11b |
| 10 | CD66b | 21 | CD197_CCR7 | 32 | CD273_PD_L2 | |  |
| 11 | TIGIT | 22 | CD11c | 33 | CD127 |  |  |

Supplemental Table 3. Antibodies involved in Immunofluorescence.

| Antibody Name | Company | Catalog No. | Region |
| --- | --- | --- | --- |
| GFP | Abconal | AE012 | China |
| FOXP3 | CST | D6O8R | USA |
| PD-1 | Abconal | A11973 | China |

Supplemental Table 4. Schmidt's score system of pancreatic histopathology.

| Pathologic changes | Scores | | | |
| --- | --- | --- | --- | --- |
|  | 0 | 1 | 2 | 3 |
| Inflammatory infiltrates | Absent | Mild | Moderate | Severe |
| Edema | Absent | Mild | Moderate | Severe |
| Parenchymal necrosis | Absent | Mild | Moderate | Severe |
| Haemorrhage | Absent | Mild | Moderate | Severe |

Supplemental Table 5. Annotated celltypes of each subgroup in Cytof.

| **Subgroup** | **Celltype** |
| --- | --- |
| C1 | NK cell |
| C2 | NK cell |
| C3 | Blood Platelet |
| C4 | Neutrophil |
| C5 | Basophilic Granulocyte |
| C6 | Neutrophil |
| C7 | Monocytes |
| C8 | Monocytes |
| C9 | Monocytes |
| C10 | Dendritic cell |
| C11 | Dendritic cell |
| C12 | Treg cell |
| C13 | central memory CD4 T cell |
| C14 | CD4+ T cell |
| C15 | Th1 cell |
| C16 | memory CD8+ T cell |
| C17 | central memory CD8+ T cell |
| C18 | CD4+ T cell |
| C19 | Tfh cell |
| C20 | activated CD8+ T cell |
| C21 | activated CD8+ T cell |
| C22 | CD8+T cell |
| C23 | Th2 cell |
| C24 | B cell |
| C25 | B cell |
| C26 | B cell |
| C27 | B cell |
| C28 | Tfh cell |
| C29 | B cell |
| C30 | Monocytes |
